# Therapeutic potentials of Quercetin in management of polycystic ovarian syndrome using Letrozole induced rat model: a histological and a biochemical study

**DOI:** 10.1186/s13048-018-0400-5

**Published:** 2018-04-03

**Authors:** Sarwat Jahan, Abira Abid, Sidra Khalid, Tayyaba Afsar, Qurat-Ul-Ain, Ghazala Shaheen, Ali Almajwal, Suhail Razak

**Affiliations:** 1 Department of Animal Sciences, Quaid-i-Azam University, Islamabad, Pakistan; 2 Department of Community Health Sciences, College of Applied Medical Sciences, King Saud University, Islamabad, Saudi Arabia; 3 Department of Biochemistry, Quaid-i-Azam University, Islamabad, Pakistan

**Keywords:** Quercetin, Metformin, Polycystic ovarian syndrome, Biochemical analysis, Histological analysis

## Abstract

**Background:**

PCOS is a leading endocrinopathy of young women instigating androgens elevation, insulin resistance, obesity, cardiometabolic and menstrual complications. The study investigated the effects of quercetin in a letrozole induced rat model of polycystic ovarian syndrome, which displayed both clinical and metabolic features as in PCOS women.

**Methods:**

Female Sprague Dawley (SD) rats were divided into four groups; control group received aqueous solution of carboxymethyl (CMC 0.5%); PCOS group administered with letrozole (1 mg/kg) dissolved in solution (CMC 0.5%); Metformin group given with metformin (20 mg/kg) + letrozole (1 mg/kg); and Quercetin group provided with quercetin (30 mg/kg) + letrozole (1 mg/kg). All doses were given orally via gavage, for 21 consecutive days and colpocytological analysis was carried till end. After 21rst day, blood was taken out, centrifuged and plasma was kept for biochemical analysis (ELISA, anti-oxidant enzymes, lipid profile) and the reproductive organs were dissected out for histopathological evaluation.

**Results:**

Quercetin as a chief member of flavonoid, showed beneficial effects by decreasing body weight, ovarian diameter, cysts and restoring healthy follicles, follicle’s extra-glandular layers, and corpora lutea in contrast to the positive control. Additionally, lipid profile and anti-oxidant status were also maintained to baseline which was very high in diseased rats (*p* < 0.001).Quercetin depicted a mark regulation in steroidogenesis by decreasing the levels of testosterone (0.78 ng/ml ± 0.14 in quercetin vs. PCOS positive control 1.69 ng/ml ± 0.17, p < 0.001) and estradiol (8.85 pg/ml ± 0.19 in quercetin vs. PCOS positive 1.61 pg/ml ± 0.29) and increasing progesterone levels (34.47 ng/ml ± 1.65 in quercetin vs. 11.08 ng/ml ± 1.17 in PCOS positive). The effects of quercetin were moderately parallel to the standard drug available in market i.e. metformin.

**Conclusion:**

The present study has confirmed that quercetin has the potentials to alleviate the hormonal and metabolic disturbances occurring in PCOS.

## Background

Polycystic ovary syndrome (PCOS) is a complex reproductive, endocrine and metabolic disorder of premenopausal women with unknown etiology. Its prevalence is 5%-20%, which is even higher in “indigenous women”, signifying the contribution of a genetic component in the pathophysiology of PCOS [1]. Rotterdam’s criteria for the diagnosis of PCOS include hyperandrogenism, ovulatory dysfunction and multiple ovarian cysts [2]. Prolonged sequelae of PCOS leads to metabolic aberrations, hyperinsulinemia, obesity, diabetes mellitus, endometrial carcinoma, amenorrhea/oligomenorrhea and cardiovascular disease [3–5].

Androgen abundance is the hallmark of this syndrome, triggered either by obesity, insulin resistance or by androgen-secreting neoplasms (Dunaif et al., 1984). Confirmed signs are hirsutism, acne, seborrhea, acanthosis nigricans and virilization. In PCOS, the co-occurrence of hyperandrogenism and hyperinsulinemia has been observed [6]. Insulin works synergistically with LH, creating an overactive androgenic state due to hyperthecosis and reduced hepatic fabrication of sex hormone binding globulin, which later endows to the anovulatory mechanisms [7].

To date, no single medication exist for PCOS, it tailors according to the symptoms. Insulin sensitizers like metformin and derivatives of thiazolidinedione are extensively used in controlling PCOS [8]. Metformin fits in the bi-guanine class of anti-hyperglycemic medicines, chemically known as N,N-dimethyl biguanide [9]. Metformin improves insulin sensitivity, impedes hepatic glucose production and decreases androgen synthesis by ovarian theca cells [10, 11]. Adjacent to benefits, metformin also possesses side issues including gastro-intestinal disturbances, lactic acidosis and renal insufficiency [9]. Therefore, it may not be a suitable drug of choice for long-term PCOS medication.

In PCOS positive subjects, alterations in the oxidant-antioxidant profile have been observed [12, 13]. However, the role of oxidative stress in the pathogenesis of PCOS can never be neglected because of its involvement in metabolic disorders like cardiovascular diseases, atherogenesis, diabetes mellitus and obesity [14]. It causes hyperplasia in ovarian mesenchyme that further contributes to pre-eclampsia, endometriosis, abortion, PCOS, lessened fertility and dysgenesis [15].

Currently, plant extracts are being widely used to treat female reproductive disorders [16, 17]. Flavonoids are plant based compounds which are of great interest due to their expansive pharmacological activities [18]. Quercetin (3,5,7,3′,4′-pentahydroxyflavone) is a flavonoid [19], ubiquitously present in fruits and vegetables especially tomato, onion, broccoli, lettuce, grapes, apple and blueberries [20]. Quercetin is among one of the most commonly consumed dietary flavonoids with an average of 25–50 mg/day [21]. It belongs to a set of plant based non-steroidal compounds known as Phyto-estrogen [19]. Quercetin is found to have antioxidant potential [22], cardiovascular protection, anticancer activity [23], anti-diabetic [24], anti-inflammatory effects and restoration of bone loss in post-menopausal women [25]. It protects low-density lipoprotein (LDL) from oxidation [26], lipid peroxidation [27] and avoids redox disproportion in cells [28]. Quercetin also modulates ovarian functions as it regulates cell steroidogenic activity and helps to correct hormonal indices [29].

Despite the presence of numerous well-known drugs, there is a need of a broad spectrum drug effective enough to cure all the symptoms of PCOS and this encourages us to focus on quercetin owing to versatile properties [9]. Hence, the present study was designed to investigate the possible effects of quercetin in reproductive, endocrine and metabolic dysfunctions in letrozole induced PCOS rat model.

## Materials and methods

### Animals

Twenty four adult female SD rats (60-70 days old and 180 ± 10 g body weight) were obtained from the Animal facility of Animal Sciences Department, Quaid-i-Azam University, Islamabad. Animals were retained in stainless steel cages (6-8 rats/cage), at room temperature 25 ± 5 °C. All the rats were kept under 10/14 h dark/light cycle and fed with standard laboratory food pellet and tap water was available ad libitum. The experiment was performed in accordance with guidelines provided by the Department of Animal Sciences, Quaid-i-Azam University, Islamabad.

### Experimental design

In this experiment, female rats showing normal estrous cycle were selected and randomly divided into four groups (*n* = 6-8); the first group served as a control and was given 0.5% aqueous solution of Carboxymethylcellulose (2 mg/kg). The second group was PCOS positive group, administered with letrozole (Femara@, Novartis, Pakistan) at the concentration of 1 mg/kg dissolved in 0.5% CMC. The third group was given with letrozole (1 mg/kg dissolved in 0.5% CMC) and metformin (2 mg/100 g body weight) which is a commercially available drug (Glucophage purchased from Merck Serono, Quetta, Pakistan) for the treatment of PCOS. Fourth group was co-treated with letrozole (1 mg/kg dissolved in 0.5% CMC) and quercetin (30 mg/kg) (Q4951 Sigma). Body weight of all groups was recorded on every 5th day. All the animals were sacrificed by decapitation on the 22nd day of treatment.

### Vaginal smear

Vaginal smears of all rats were collected daily before treatment by using dropper filled with normal saline (0.9%, NaCl). A hematoxylin staining was done and stages were evaluated microscopically (Nikon, 187,842, Japan) as purposed by Shorr, 1941 [30].

### Blood and tissue sampling

After 21 days of treatment, animals were decapitated at diestrus stage. Trunk blood was collected and centrifuged at 3000 rpm for 15 min. Plasma was separated and stored at − 20 °C until analyzed for biochemical and hormonal analysis. Ovaries were cleaned in saline and made fat free. Right ovary was stored at − 80 °C for the determination of antioxidant status while the left ovary was fixed in 10% formalin and processed for histology.

### Ovarian histology & Histomorphological examination

The excised ovaries were fixed in 10% formalin and processed further as a routine histological procedure, they were serially sectioned at 5 μm thickness using microtome (Thermo, Shandon finesse 325, UK) and every 20th section was placed on a glass slide. Ten representative sections per rat ovary were chosen so that follicles were not repeated. The slides were stained with hematoxylin and eosin using a standard protocol. For morphometric analysis, the diameter of the largest follicle consisting of a clear oocyte was taken. The thickness of granulosa and theca cells was measured using image J2x software. Follicles were counted in each section and classified according to the work of Luo et al., 2008 [31].

### Estimation of antioxidant enzymes

Ovarian tissue (20 mg) was homogenized in 2 ml of phosphate buffer (pH 7.4) and centrifuged at 12000 rpm for 30 min at 4 °C. Supernatant was collected to determine the antioxidant status of following assays: Catalase (CAT) was estimated by the method developed by Chance and Maehly [32]. After one minute at a wavelength of 240 nm, variations in absorbance of solution were noted. Absorbance change of 0.01 units in one minute was called one unit of CAT activity. Peroxidase activity (POD) was determined by following the scheme of Chance and Maehly. Changes in absorbance were noted at 470 nm after an interval of one minute. Absorbance change of 0.01 units in one minute was called as one unit of POD activity. Superoxide dismutase (SOD) activity was assessed by method purposed by Kekkar et al. [33]. After 1 min of reaction, readings were noted at 560 nm and results were explained as units/mg of protein. By using the procedure of Carlberg and Mannervik (1975) with some modifications, glutathione reductase levels were evaluated. NADPH activity was noted at 25 °C and at a wavelength of 340 nm and expressed as nmol of NADPH oxidized/min/mg protein with the help of coefficient of molar extinction of 6.22 × 103 M-1 cm-1 [34]. For thiobarbituric acid reactive substances (TBARS), a method developed by Wright et al. and Iqbal et al. was used to assess lipid peroxidation. At wavelength 535 nm, readings were noted from spectrophotometer [35, 36]. Results were explained as μmol of TBARS/min/mg tissue at 37 °C with a coefficient of molar extinction of 1.56 × 105 M-1 cm-1.

### Lipid profile assay

Fasting glucose, HDL-C, LDL-C, total cholesterol and triglyceride levels were quantified by following the protocols provided with kits (Merck) on Piccos 05 chemistry analyzer.

### Hormonal analysis

The concentration of plasma estrogen, progesterone and testosterone were measured via Enzyme Linked Immuno Sorbent Assay (ELISA), with the help of commercial kits (ELISA kit, Amegnix, Inc., Burlingame, CA, USA) and the procedure was followed as given in the kit catalog. The concentration of estrogen, progesterone and testosterone were estimated from the standard curve.

### Statistical analysis

All values were expressed as mean ± SEM. Data analysis was done by using analysis of variance (ANOVA) and Tukey’s post hoc test for multiple comparisons. The inter-assay CV was < 15% and intraassay CV was < 10% for biochemical tests. The values for *P* < 0.05 were considered significant. Data were analyzed using Graph Pad Prism version 5.00.

## Results

### Effect on body weight and estrous cycle

PCOS induced rats showed 24% increase in mean body weight as compared to control group at the end of the experiment. Contrarily, both quercetin and metformin-treated groups did not depict any mark difference in mean body weight when compared with control group (Table 1).

**Table 1:** Mean ± SEM of Body Weight, Glucose level, Body mass index, Ovarian diameter, Ovarian weight, Diameter (μm), Peripheral granulosa layer thickness (μm), Theca layer thickness (μm) of secondary, tertiary and cystic follicle in Control

Parameters	Control	PCOS	PCOS + Metformin	PCOS + Quercetin	
Initial Body Weight (gm)	180.50 ± 1.82	180.33 ± 3.92	180.42 ± 1.85	180.14 ± 1.54	
Final Body Weight (gm)	199.25 ± 2.19	223.16 ± 3.85	193.51 ± 2.01	199.28 ± 2.75	
Glucose (mg/dL)	56.62 ± 0.48	72.20 ± 0.58 ^a***^	56.22 ± 0.33 ^b***^	56.14 ± 0.29 ^b***^	
Ovary diameter (mm)	28.50 ± 1.08	68.16 ± 3.12 ^a***^	29.72 ± 1.06 ^b***^	33.85 ± 0.55 ^b***^	
Ovary weight (mg)	3.63 ± 0.14	5.23 ± 0.11 ^a***^	3.79 ± 0.07 ^b***^	3.91 ± 0.10 ^b***^	
Secondary follicle diameter (μm)	236.96 ± 15.06	270.42 ± 13.84	231.15 ± 14.94 ^b*^	217.72 ± 12.55 ^b*^	
Granulosa layer thickness (μm)	46.17 ± 1.39	50.83 ± 3.14	41.33 ± 1.06	41.83 ± 2.01	
Theca layer thickness (μm)	20.43 ± 1.22	21.82 ± 0.80	22.18 ± 1.02	21.02 ± 0.73	
Tertiary follicle diameter (μm)	419.10 ± 14.04	471.12 ± 15.77	414.22 ± 12.42	402.58 ± 15.48	
Granulosa layer thickness (μm)	52.34 ± 3.38	33.43 ± 6.10 ^a**^	53.53 ± 1.68 ^b**^	49.14 ± 2.20 ^b*^	
Theca layer thickness (μm)	25.01 ± 2.08	21.96 ± 1.32	23.95 ± 0.72	22.75 ± 0.88 ^a*^	
Cystic follicle diameter (μm)	0 ± 0	605.57 ± 7.61 ^a***^	479.29 ± 20.77 ^b***^	527.66 ± 26.05 ^b**^	
Granulosa layer thickness (μm)	0 ± 0	30.39 ± 1.22 ^a***^	32.54 ± 3.20 ^a***^	36.90 ± 3.61 ^a***b*^	
Theca layer thickness (μm)	0 ± 0	24.64 ± 0.90 ^a***^	23.20 ± 0.52 ^a***^	27.32 ± 2.31 ^a***^	

PCOS, PCOS+Metformin, PCOS+Quercetin treated groups after 21 days of experiment

Values are expressed as Mean ± SEM

*, **, *** indicate significance from the control group at *P* < 0.001, 0. 01 and 0.05 probability level

**Fig. 1:**
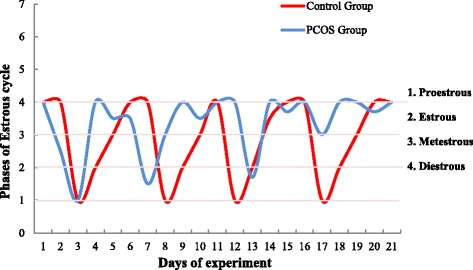
Representation of estrous cycle of control and PCOS group for 21 consecutive days of experiment

A normal estrous cycle of 4-5 days with all four phases in sequential order was observed in the control group, whereas it was completely disrupted in PCOS induced rats with a dominant diestrus stage. Quercetin and metformin-treated groups showed restoration of the estrous cycle (Fig. 1).

### Ovarian weight and diameter changes

The weight and diameter of the ovary were 68.16 mg ± 3.12 and 5.23 mm ± 0.11respectively in cystic rats versus control group 28.50 mg ± 1.08 and 3.63 mm ± 0.14. The metformin and quercetin-treated rats showed a significant decrease (*P* < 0.001) in ovarian diameter as compared to untreated PCOS group (Table 1).

### Morphometrical analysis

PCOS positive rats showed numerous large-sized cysts instead of secondary and tertiary follicles. However, quercetin treatment displayed significant (*P* < 0.01) decrease in cystic follicle diameter, unlike PCOS group. However, the decrease was much pronounced in metformin-treated groups in contrast to cystic rats (*P* < 0.001) (Table 1).

### Thickness of peripheral granulosa and theca layer

**Fig. 2:**
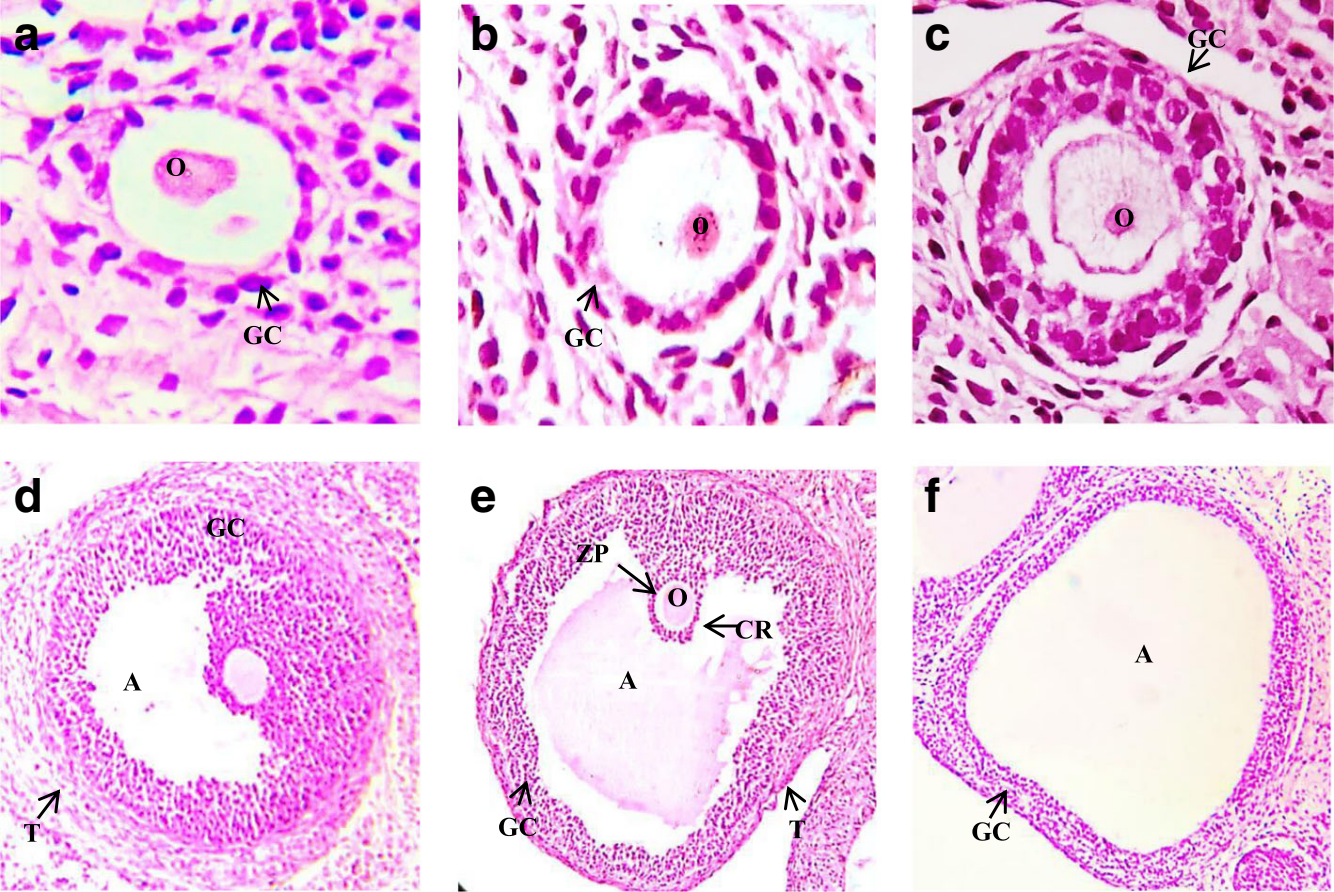
Photomicrographs of different ovarian follicles in control and PCOS rats. **a** Primordial follicle with flattened layer of granulosa cells (GC) at (× 40) with oocyte (O) **b** Primary follicle with cuboidal layer of granulosa cells (GC) and a well-defined oocyte (O) at (× 40) **c** Secondary follicle with 2-3 layers of granulosa cells (GC), follicular fluid and a prominent oocyte (O) at (× 40) **d** A tertiary follicle with clear antrum (A), thick granulosa cell layer (GC) surrounded by theca (T) layers at (× 20) **e** A graffian follicle with a large antrum (A), a primary oocyte (O) surrounded by zona pellucida (ZP) and corona radiate (CR) and thick graulosa layer along with theca layer (T) at (× 20) (**f**) A cystic follicle with large antrum (A) lacking oocyte and decreased granulosa layer (GC)

The thickness of granulosa and a thecal layer of secondary follicles in PCOS induced group showed an increase and the treated groups showed a decrease, also the change was negligible. The wideness of granulosa layer in tertiary follicles in diseased control exposed substantial decrease (*P* < 0.001) discrepant to control. However, metformin and quercetin treatment reversed these values near to normal ones (*P* < 0.001 & *P* < 0.05 respectively). The metformin group presented a minimal increase in granulosa and theca layer thickness of cystic follicles when matched with untreated PCOS group. Quercetin treatment was also effective and normalized the thickness of theca and granulosa layer significantly (*P* < 0.05) (Table 1, Fig. 2).

### Number of ovarian follicles

**Fig. 3:**
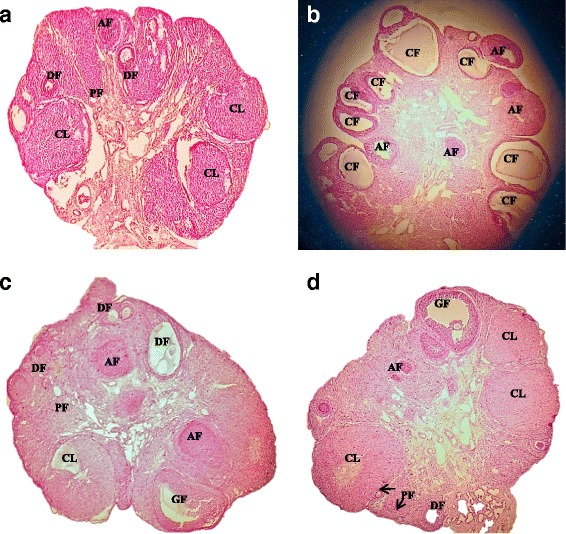
Photomicrographs of representative ovarian cross section from control, PCOS, Metformin and Quercetin treated rats after 21 days of experiment. **a** Control with different stages of ovarian follicles including primary follicles (PF), developing follicles (DF), corpus luteum (CL) and atretic follicle. **b** PCOS rat ovary cross-section containing various cystic follicles (CF) and atretic follicles (AF). **c** Metformin-treated rat ovary with primary follicles (PF), developing follicles (DF), atretic graffian follicle (GF), corpus luteum (CL) and atretic follicle (AF). **d** Quercetin treated rat ovary containing primary follicles (PF), developing follicle (DF), a healthy graffian follicle (GF), corpus luteum (CL) and atretic follicle (AF)

A slight decrease was observed in a mean number of primordial and primary follicles of PCOS induced group as compared to the control group. But, metformin and quercetin treatment reversed the number of these follicles, considerably (*P* < 0.05). The mean number of secondary (*P* < 0.05), tertiary (*P* < 0.001) and graffian follicles were declined in PCOS positive rats dissimilar to the control group, however, the co-treatment of metformin and quercetin has remarkably increased these follicles number (*P* < 0.01). Similarly, clear increase (*P* < 0.001) in cystic and atretic follicles while a decrease in corpus luteum was perceived in PCOS positive group that was upturned by treatment of metformin and quercetin (*P* < 0.001), indicating the recovery of the syndrome (Table 2, Fig. 3).

**Table 2:** Mean ± SEM number of primordial, primary, secondary, tertiary, graffian, cystic, atretic follicle and corpus luteum (5 sections per ovary) in control, PCOS, PCOS + Metformin and PCOS +Quercetin treated adult female rats after 21 days of treatment

Developing follicles (μm)	CONTROL	PCOS	PCOS + METFORMIN	PCOS + QUERCETIN	
Primodial	7.80 ± 0.80	7.33 ± 0.38	8.14 ± 0.47	7.85 ± 0.42	
Primary	6.80 ± 0.33	5.50 ± 0.20	6.85 ± 0.40 ^b*^	6.90 ± 0.31 ^b*^	
Secondary	2.00 ± 0.89	0.60 ± 0.15 ^a*^	2.14 ± 0.24 ^b**^	2.00 ± 0.53 ^b*^	
Tertiary	1.61 ± 0.22	0.16 ± 0.15 ^a***^	1.30 ± 0.17 ^b**^	1.28 ± 0.17 ^b**^	
Graffian	1.00 ± 0.63	0.16 ± 0.15	0.71 ± 0.17	0.85 ± 0.13	
Cystic Follicle	0 ± 0	11.33 ± 0.45 ^a***^	1.57 ± 0.18 ^a**b***^	1.60 ± 0.20 ^a**b***^	
Atretic Follicle	2.80 ± 0.33	8.00 ± 1.82 ^a***^	3.28 ± 0.26 ^b***^	3.00 ± 0.20 ^b***^	
Corpus Luteum	3.60 ± 0.35	1.66 ± 0.20 ^a***^	3.57 ± 0.18 ^b***^	3.43 ± 0.18 ^b***^	

Values are expressed as Mean ± SEM.*, **, *** indicate significance from the control group at *P* < 0.001, 0.01 and 0.05 probability level

^a^Value vs control, ^b^Value vs PCOS

### Antioxidant enzymes status

A noticeable decrease (P < 0.05 and *P* < 0.001) in total protein content, CAT, SOD, POD and GR levels were realized in ovarian tissue of letrozole-treated rats as compared to control. Quercetin and metformin treatment reversed these values near to baseline. A significant increase in TBARS level was observed in PCOS induced group that was decreased by the action of metformin and quercetin (*P* < 0.01) (Table 3).

**Table 3:** Mean ± SEM of total Protein, CAT, SOD, POD, T-BARS, GR, Cholesterol, Triglycerides, HDL-C, LDL-C and VLDL-C, Testosterone, Progesterone and Estradiol Concentrations in Control, PCOS, PCOS+Metformin and PCOS+Quercetin treated female rats after 21 Days of experiment

Parameters	Control	PCOS	PCOS + Metformin	PCOS + Quercetin	
Total Protein (mg/g)	33.62 ± 0.52	27.22 ± 1.39 ^a*^	31.50 ± 1.53	30.65 ± 0.95	
CAT (u/mg)	33.64 ± 1.38	5.26 ± 0.12 ^a***^	30.71 ± 0.94 ^b***^	40.74 ± 0.75 ^a** bc***^	
SOD (u/mg)	8.14 ± 0.14	5.26 ± 0.12 ^a***^	6.79 ± 0.13 ^a** b*^	9.16 ± 0.21^a** bc***^	
POD (μM/min)	0.47 ± 0.02	1.25 ± 0.07 ^a***^	0.98 ± 0.04 ^a***b*^	0.72 ± 0.04 ^a*b*** c*^	
TBARS (μmol/mg)	23.74 ± 0.34	32.28 ± 2.10 ^a**^	30.83 ± 0.74 ^a*^	25.10 ± 1.14 ^b*^	
GR (nmol/mg protein)	1.42 ± 0.03	1.20 ± 0.02 ^a***^	1.27 ± 0.02^b**^	1.39 ± 0.02 ^b**^	
Cholesterol (mg/dl)	55.41 ± 0.56	63.96 ± 0.69 ^a***^	55.84 ± 0.80 ^b***^	55.61 ± 1.26 ^b***^	
Triglycerides (mg/dl)	51.93 ± 0.63	68.06 ± 1.42 ^a***^	58.60 ± 0.65 ^b***^	56.07 ± 0.78 ^b***^	
HDL-C (mg/dl)	28.47 ± 0.18	22.19 ± 1.00 ^a***^	25.74 ± 0.17 ^a*b**^	23.97 ± 0.11 ^a***^	
LDL-C (mg/dl)	16.55 ± 0.51	26.95 ± 1.67 ^a***^	20.37 ± 1.00 b^*^	19.63 ± 1.14 ^b**^	
VLDL-C (mg/dl)	10.39 ± 0.13	13.73 ± 0.98 ^a*^	11.72 ± 0.13	9.89 ± 0.27 ^b*^	
LDL-C/HDL-C	0.58 ± 0.02	1.63 ± 0.03 ^a***^	0.72 ± 0.02 ^a* b***^	0.63 ± 0.03 ^b***^	
TC/HDL-C	1.95 ± 0.02	3.36 ± 0.03 ^a**^	2.17 ± 0.03 ^b**^	1.72 ± 0.03 ^b***^	
TG/HDL-C	1.82 ± 0.02	2.69 ± 0.36	2.32 ± 0.06	2.15 ± 0.16	
Testosterone (ng/ml)	0.38 ± 0.02	1.69 ± 0.17 ^a***^	0.52 ± 0.11 ^b***^	0.78 ± 0.14 ^b***^	
Progesterone (ng/ml)	39.65 ± 2.28	11.08 ± 1.17 ^a***^	33.87 ± 2.96 ^b***^	34.47 ± 1.65 ^b***^	
Estradiol (pg/ml)	9.29 ± 0.32	1.61 ± 0.29 ^a***^	9.24 ± 0.59 ^b***^	8.85 ± 0.19 ^b***^	

Values are expressed as Mean ± SEM

*, **, *** indicate significance from the control group at *P* < 0.001, 0.01 and 0.05 probability level

^a^Value vs control, ^b^Value vs PCOS and ^c^Value vs PCOS+Metformin

### Lipid profile

#### Glucose, cholesterol and triglycerides

In PCOS induced group, a significant increase (*P* < 0.001) in glucose, cholesterol and triglyceride levels was noticed compared to control group. However, treatment with metformin and quercetin significantly decreased (*P* < 0.001) their levels unlike PCOS induced group (Tables 1, 3).

#### HDL-C, LDL-C and VLDL

There was a remarkable increase (P < 0.001) in LDL-C and VLDL-C in PCOS group compared to control group, whereas HDL-C levels were considerably decreased in PCOS group. Treatment with metformin and quercetin lessened HDL-C and VLDL-C and HDL-C in comparison to the PCOS positive animals (Table 3).

#### LDL-C/HDL-C, TC/HDL-C and TG/HDL-C

Similarly, a profound increase (P < 0.001) in ratios of LDL-C/HDL-C, TC/HDL-C and TG/HDL-C were observed in PCOS whereas metformin and quercetin-treated groups significantly prevented the increase (Table 3).

#### Hormonal assay of PCOS induced and treated rats:

A significant (*P* < 0.001) decrease in the levels of progesterone and estradiol, while an increase in testosterone was observed in PCOS induced rats when compared to control. However, treatment with metformin and quercetin prodigiously optimized these values (*P* < 0.01) (Table 3).

## Discussion

The present study successfully exhibited the development of PCOS rat model by following the method of Kafali et al., 2004. Letrozole, chemically designated as 4,4′-(1H-1,2,4-Triazol-1-ylmethylene) dibenzonitrile, a non-steroidal aromatase inhibitor, suppresses whole body aromatization, paracrine signaling, folliculogenesis and ovarian function [37, 38]. Adult rats treated with letrozole for 3 weeks developed features very much similar to women PCOS. Vaginal smear histology is a key indicator of ovarian physiology. PCOS positive rats were almost acyclic, specifying the presence of cysts contrary to vehicle [39]. Moreover, this model also showed a remarkable increase in body weight [6]. Quercetin treated rats showed a significant drop in weight that justified quercetin’s ability to downregulate the genes responsible for adiposity [40]. Besides weight gain, excessive hair growth is also a clue for PCOS, attributed to glut androgens production [6]. On the basis of our observation, we could say that quercetin treatment was successful in subsiding hirsutism.

Quercetin is a plant-centered polyphenolic compound, most distinguished agent for treating metabolic comorbidities [41]. Insulin and inflammatory signaling pathway have close cross talks which turned out to insulin resistance in PCOS [42]. The ovaries from PCOS induced group looked larger in size, reddish in color with bulgy appearance unlike that of the control levels of pro-inflammatory markers i.e. TNFα and IL-6 by inhibiting Toll-like receptor 4, indicated by Wang et al., 2017 [43]. Levels of glucose were very high in PCOS positive group that standardizes in treated groups, convincing the ability of Quercetin to maintain glucose homeostasis [44]. Rezvan et al., 2017 work depicted that quercetin stimulates glucose uptake by activating AMPK-dependent and insulin-independent pathways to increase GLUT-4 content [45]. Further, it downregulates the key enzymes for gluconeogenesis and protects the β-cell function of islet [46].

Aside from inflammation and hyperglycemia, oxidative stress also contributes to PCOS [47].Oxidative stress is defined as an imbalance in normal cell milieu due to overproduction of reactive oxygen species (free radical) and inadequate anti-oxidant defense. Excessive ROS is produced subsequent to oxygenase reactions, electron transport reaction in mitochondria and Super oxide (SO) anion reaction in cytochrome P450 [48]. Mitochondria is a fundamental organelle of the cell, any dysfunction can alter the generation of adenine triphosphate (ATP), crucial for gametogenesis which otherwise causes cell cycle cessation and cellular injury i.e. DNA damage and cell death [49]. Superoxide dismutase helps in detoxifying SO anion by converting it to H2O2. Catalase and glutathione peroxidase (GPx) further scavenges the resultant products to water (H2O) [50]. In PCOS positive group, disproportion in the amount of SOD, CAT,POD,GR and GSHPX was perceived, our findings also co-related with the previous research [51]. Quercetin administration has counterbalanced the ROS levels and improved the antioxidant activities by inhibition of NADPH oxidases.

Thiobarbituric acid reactive substances (TBARS) is the best common marker used to determine the index of lipid peroxidation [52]. Lipid peroxidation is described as an oxidative deprivation of lipids, initiating a free radical chain reaction of polyunsaturated fatty acids of the fatty acid membrane [53]. Diseased control showed dyslipidemia i.e. lessened HDL-C and more levels of plasma triglycerides, LDL-C, TBARS and total cholesterol [54]. Dyslipidemia is a root cause of coronary artery disease in PCOS patients [16, 55]. Quercetin protects cellular injury directly by scavenging free radicals and prevents from atherosclerosis by inhibiting LDL from oxidation, our data has also verified it [27]. Moreover, it has decreased cholesterol and increased HDL-C levels and metformin has the parallel results [56].

The hormonal statistics of letrozole induced rats embodied a hyper-androgenized state responsible for abnormal ovarian physiology [18]. The disruption in usual hypothalamic pituitary gonadal axis, elevate both testosterone and LH and developed a disease state [57, 58]. LH excites testosterone secretion in a thecal layer of follicles by prompting PI3K/Akt pathway [59]. The PI3K pathway is a mediator of LH dependent Akt phosphorylation in follicles, uphill the expression of ovarian CYP17A1 gene which further increases the activity of 17-a hydroxylase enzyme [60, 61]. This 17α-hydroxylase is a core enzyme which catalyzes the steroidogenic transformation of progesterone to androgens, thus raising the level of androgens. Present study and work of Shah et al., 2015 gave an evidence that quercetin exhibited anti-androgenic potentials by completely obstructing PK13 pathway and downregulating CYP17A1 gene [29]. Conversely, metformin did not show remarkable downregulation of CYP17A1 gene, which means it might control ovarian steroidogenesis by regulating insulin levels [29]. Administration of Quercetin is beneficial in preserving levels of testosterone, estrogen and progesterone.

Histology is a pre-eminent aspect to assess ovarian changes. The ovaries of PCOS induced rats exhibited numerous enlarged cysts, lacking an oocyte, granulosa and theca layer hyperplasia and increased follicular atresia, also well-matched with previous studies [51, 39]. The bare existence of corpus luteum spectacled an anovulatory state that makes chances of conception minimal [62]. Corpus luteum is essential for the synthesis of progesterone hormone which controls the reproductive cycles and prepares the uterus for implantation if conception happened [63]. The decreased number of secondary and tertiary follicles evident the overproduction of androgens that impedes with normal follicular maturation process, in diseased group [51]. Contrarily, the metformin and quercetin-treated groups showed a marked recovery of ovarian tissue with the appearance of developing and antral follicles, prominent reduction in cysts and regular luteinization [51]. Also, their follicles showed prominent and clear antrum with no cellular debris. The ovarian cortex of both treated groups displayed the proliferation of numerous healthy follicles along with improved vascularization of the thecal layer. Presence of more corpora lutea in the cured groups indicated renovation of estrous cycle to normal functioning [51].

Further investigations are required to assess the concentration of quercetin in blood and its bioavailability. This study is limited to a single dose of quercetin; however, a dose-dependent study should be conducted in order to observe the effects of different dosages in the treatment of PCOS.

## Conclusion

Our findings showed that quercetin is a powerful flavonoid with the ability to combat metabolic and endocrine comorbidities in PCOS. Quercetin presented beneficial effects on lipid profile, hormonal indices, and glucose uptake. Also, it exerted strong anti-oxidant potentials and recovered ovarian cysts, healthy follicles and regained the pleura of follicles. The restoration of ovarian function and anti-androgenic potentials of quercetin may offer an advantageous remedy for PCOS. Furthermore, direct studies on humans are desired to investigate the therapeutic potentials, so that quercetin can be used as an adjunct therapy or alone as a curative of PCOS.
